# The Coagulation of Blood in Arteries by Means of Solution of Perchloride of Iron—Aneurism of the Suborbital Artery Cured by This Means

**Published:** 1853-07

**Authors:** 


*The Coagulation of Blood in Arteries by Means of Solution of Perchloride of Iron—Aneurism of the Suborbital Artery Cured by this Means.*—Our readers, no doubt, remember the interesting account communicated by M. Lallemand to the Academy of Sciences, of the experiments made by M. Pravas of Lyons. This question having been lately brought before the Surgical Society of Paris, connected with its successful application in man, we will briefly detail in what M. Pravas's method consists.

He makes use of perchloride of iron at the maximum of concentration, a few drops of which it is to be injected into the vessel in which it is desirous to obtain coagulation. This injection is to be made with a very fine trocar, of either gold or platinum, the diameter of which is scarcely greater than that of a needle; this is to be introduced very obliquely, through the parietes of the artery, with a wriggling motion. To this trocar is adjusted a small syringe, the piston of which works with a rack and pinion motion, so that the injection proceeds without impulse, drop by drop, and in such a way, the quantity of liquid injected can be accurately measured. Every turn of the pinion allows of the escape of two drops of the liquid. Whilst this injection is used, the circulation is momentarily arrested in the vessel by means of pressure above and below. A few drops suffice (three or four in a sheep, six or eight in the horse) to form a solid and resisting clot.

Up to this time, the experiments had been only made on animals, but M. Lallemand expressed a hope to see them succeed in man. This appeal has been heard. M. Raoul Deslongchamps has just sent before the Surgical Society of Paris a case of aneurism of the suborbital artery treated with success by injection of perchloride of iron. It was a tumor situated in the suborbital region, affording pretty strong pulsations, isochronous with the heart's action. This tumor, for the cure of which compression had been used without effect, disappeared completely by means of the injection, as recommended by M. Pravas, after presenting some inflammatory symptoms, which easily yielded to antiphlogistic treatment.—*Dub. Med. Press,* from *Gazette Medicale de Toulouse* and *Presse Medicale Belge.*

